# Cognition in chronic kidney disease: a systematic review and meta-analysis

**DOI:** 10.1186/s12916-016-0745-9

**Published:** 2016-12-14

**Authors:** Israel Berger, Sunny Wu, Philip Masson, Patrick J. Kelly, Fiona A. Duthie, William Whiteley, Daniel Parker, David Gillespie, Angela C. Webster

**Affiliations:** 1 Sydney School of Public Health, Sydney Medical School, University of Sydney, Sydney, Australia; 2 University of Edinburgh, Edinburgh, UK; 3 Behavioral Health, Desert AIDS Project, Palm Springs, CA USA; 4 Centre for Transplant and Renal Research, Westmead Millennium Institute, University of Sydney at Westmead, Sydney, Australia

**Keywords:** Renal insufficiency, Chronic kidney disease, Mild cognitive impairment, Dementia, Psychometrics, Cognition, Neuropsychological tests

## Abstract

**Background:**

Cognitive impairment is common in people with chronic kidney disease (CKD) and associated with increased morbidity and mortality. Subtle changes can impact engagement with healthcare, comprehension, decision-making, and medication adherence. We aimed to systematically summarise evidence of cognitive changes in CKD.

**Methods:**

We searched MEDLINE (March 2016) for cross-sectional, cohort or randomised studies that measured cognitive function in people with CKD (PROSPERO, registration number CRD42014015226). The CKD population included people with eGFR < 60 mL/min/1.73 m^2^, not receiving renal replacement therapy, in any research setting. We conducted a meta-analysis using random effects, expressed as standardised mean differences (SMD) with 95% confidence intervals (CI). Outcomes were performance in eight cognitive domains. Bias was assessed with the Newcastle-Ottawa Scale (NOS).

**Results:**

We identified 44 studies reporting sufficient data for synthesis (51,575 participants). Mean NOS score for cohort studies was 5.8/9 and for cross-sectional 5.4/10. Studies were deficient in NOS outcome and selection due to poor methods reporting and in comparison group validity of demographics and chronic disease status. CKD patients (eGFR < 60 mL/min/1.73 m^2^) performed worse than control groups (eGFR ≥ 60 mL/min/1.73 m^2^) on Orientation & Attention (SMD –0.79, 95% CI, –1.44 to –0.13), Language (SMD –0.63, 95% CI, –0.85 to –0.41), Concept Formation & Reasoning (SMD –0.63, 95% CI, –1.07 to –0.18), Executive Function (SMD –0.53, 95% CI, –0.85 to –0.21), Memory (SMD –0.48, 95% CI, –0.79 to –0.18), and Global Cognition (SMD –0.48, 95% CI, –0.72 to –0.24). Construction & Motor Praxis and Perception were unaffected (SMD –0.29, 95% CI, –0.90 to 0.32; SMD –1.12, 95% CI, –4.35 to 2.12). Language scores dropped with eGFR (<45 mL/min/1.73 m^2^ SMD –0.86, 95% CI, –1.25 to –46; 30 mL/min/1.73 m^2^ SMD –1.56, 95% CI, –2.27 to –0.84). Differences in Orientation & Attention were greatest at eGFR < 45 mL/min/1.73 m^2^ (SMD –4.62, 95% CI, –4.68 to –4.55). Concept Formation & Reasoning differences were greatest at eGFR < 45 mL/min/1.73 m^2^ (SMD –4.27, 95% CI, –4.23 to –4.27). Differences in Executive Functions were greatest at eGFR < 30 mL/min/1.73 m^2^ (SMD –0.54, 95% CI, –1.00 to –0.08).

**Conclusions:**

Cognitive changes occur early in CKD, and skills decline at different rates. Orientation & Attention and Language are particularly affected. The cognitive impact of CKD is likely to diminish patients’ capacity to engage with healthcare decisions. An individual’s cognitive trajectory may deviate from average.

**Electronic supplementary material:**

The online version of this article (doi:10.1186/s12916-016-0745-9) contains supplementary material, which is available to authorized users.

## Background

Cognitive impairment is cognitive decline greater than expected with normal ageing but which does not interfere notably with activities of daily living. Poor cognitive function has been linked to poor health literacy, poorer medication adherence, worse physical and mental health, and greater morbidity and mortality. Chronic kidney disease (CKD) may be an independent risk factor for cognitive impairment. A recent systematic review by Etgen et al. [1] found that, in cross-sectional and longitudinal studies, cognitive impairment and incident cognitive impairment, respectively, were more common in CKD patients compared to people without CKD. Heterogeneity between studies was high, and age and sex were significant contributors. Six cross-sectional and six longitudinal studies could be included in their meta-analysis. While there were few included studies, there was no evidence of publication bias, and their data suggest that lower estimated glomerular filtration rate (eGFR) may be associated with greater incidence of cognitive impairment [1]. Although the economic burden of mild cognitive impairment and CKD-related cognitive changes is poorly understood, the cost of dementia has been studied in detail. Lower neuropsychological test scores are associated with increased social and financial costs, including caregiver burden [2–4].

In clinical practice, screening for or monitoring of cognitive impairment relies on the use of cognitive tests. For many clinicians, ease of use and quick administration of cognitive tests may be particularly important. Brief cognitive screening tests, such as the Mini-Mental State Examination (MMSE), have been popular as a result [5]. However, general screening tools may not differentiate specific aspects of cognition that are most affected. Cognition is classified into several discrete domains, encompassing such diverse processes as visuo-spatial perception, auditory memory, visual memory, attention span, motor function, and mathematical reasoning.

The pattern of cognitive impairment in CKD is not clear. With dialysis, CKD-related cognitive impairment is at least partially reversible (with the domains of Orientation & Attention and Memory showing significant improvement), and all domains show improvement with renal transplantation [6, 7]. It is important to understand the pattern of cognitive impairment in CKD to distinguish it from neurodegenerative diseases, stroke and traumatic brain injury, which can co-exist with CKD, may be related to the aetiology of particular patients’ kidney disease, and may be treatable if properly diagnosed. Unlike CKD-related cognitive impairment, these conditions tend to present as acute events with significant deficits specific to the anatomy affected and which may be amenable to specific cognitive or occupational therapies aimed at enhancing function rather than reversing the cause [8–10]. CKD patients are at higher risk than the general population for stroke, and the likelihood of stroke increases with falling eGFR [11]. Nevertheless, CKD-related cognitive impairment can also be severe, and patients may become unable to make healthcare decisions [12]. Identifying the pattern of CKD-related cognitive impairment enables two steps toward improving care for CKD patients. Firstly, by specifying the phenomenon under investigation, it may bring us closer to identifying potential mechanisms, anatomical areas affected, and treatments. Secondly, by identifying CKD-related deficits, it may enable recommendations for shared decision-making, advance care planning, and addressing self-management challenges related to the complex care needs of CKD patients before their decision-making capacity diminishes.

We aimed to systematically review patterns of cognitive impairment in CKD, specifically the cognitive domains most affected, and to investigate any change with declining kidney function.

## Methods

### Protocol and registration

The study protocol was registered with the international prospective register of systematic reviews (PROSPERO, registration number CRD42014015226). Methodology is reported according to Meta-analysis of Observational Studies in Epidemiology (MOOSE) criteria.

### Eligibility criteria, information sources, and search strategies

We included all randomised controlled trials, cohort studies and cross-sectional studies where cognitive function was measured and comparisons were possible between non-CKD and CKD patients and/or between groups with different stages of CKD. We did not make comparisons with any groups on renal replacement therapy. For studies that also had dialysis or transplant groups, only control groups (eGFR ≥ 60 mL/min/1.73 m^2^) and groups with eGFR < 60 mL/min/1.73 m^2^ on conservative (non-dialytic) treatment were included in this review. Case studies, case series, studies of children, and studies of patients currently being treated for acute kidney injury (AKI) were excluded.

We searched MEDLINE (inception to March 2016) using an optimally sensitive search strategy developed by a specialist librarian (Additional file 1), without language restriction.

### Study selection, data collection, and risk of bias appraisal

Two reviewers (SW, FAD) independently screened titles and abstracts of reports to identify potentially eligible studies. Where necessary, full text was retrieved to determine whether a study satisfied the inclusion criteria.

Two reviewers (SW, IB) independently extracted data from included studies using a standardised electronic form, and disagreements were resolved through discussion. Where more than one publication for a study was retrieved, reports were grouped with each report reviewed in full to ensure inclusion of all relevant data. When repeated measures were collected, we recorded only the baseline measures to avoid measuring learning effects or effects of treatment. We attempted to contact authors of those studies published from 2000 to present that reported insufficient data for meta-analysis to get more information.

We assessed risk of bias using the Newcastle-Ottawa Scale (NOS) for cross-sectional and cohort studies [13, 14]. The NOS is a tool to assess bias in studies where each bias-reducing criterion is used to award stars: selection of participants (maximum of four stars for cohort and five for cross-sectional), comparability of groups (maximum of two stars), and measurement (maximum of three stars). We calculated inter-rater reliability for the two reviewers using only English-language articles, using a kappa statistic. We also evaluated appropriateness of actual comparison groups (not statistical adjustments) using two indicators: demographic variables and chronic disease state. Studies that did not report sufficient participant demographic or disease state data were deemed inappropriately matched. Studies with appropriate comparison groups based on demographic variables reported similar (as defined by *p* > 0.05) age, ethnicity, education, and sex (K = 1.0). Studies with appropriate comparison groups based on chronic disease states recruited participants from similar settings (e.g. both groups were hospitalised) and compared CKD participants to non-CKD participants with similar chronic disease states involving fatigue and disability (K = 0.97). We used funnel plots to examine the risk of publication bias.

### Summary measures and synthesis of results

Multiple neuropsychological tests exist that are used to test similar or the same aspects of cognition, thus making analysis at the test level inadequate for examining the pattern of cognitive impairment [15]. Two psychologists (DP, IB) independently categorised all cognitive tests according to widely accepted neuropsychological testing categories; Orientation & Attention, Perception, Memory, Language, Construction & Motor Praxis, Concept Formation & Reasoning, and Executive Functions. Ambiguities we resolved through discussion. Tests that measured multiple domains in brief, e.g. the MMSE, were assigned to the domain of Global Cognition.

We summarised results across cognitive domains using a random effects model expressed as standardised mean differences (SMD) with 95% confidence intervals (CI). All SMDs were analysed such that lower scores indicated poorer outcome in CKD patients (i.e. negative, favouring non-CKD, and positive, favouring CKD). CKD patients for the purpose of this review were defined as non-dialysed patients with eGFR less than 60 mL/min/1.73 m^2^. Stages 1 and 2 CKD (eGFR ≥ 60 mL/min/1.73 m^2^) were conceptualised alongside non-CKD for analysis. Stage 3A was defined as eGFR 45–59 mL/min/1.73 m^2^, 3B as eGFR 30–44 mL/min/1.73 m^2^, 4 as eGFR 15–29 mL/min/1.73 m^2^, and 5 as eGFR < 15 mL/min/1.73 m^2^. In addition, we performed sub-group analyses stratified by eGFR [16]. Where studies reported multiple tests that mapped to the same cognitive domain, we synthesised overall scores using a validated method to account for the correlation among multiple tests of the same domain, assuming an among-test correlation coefficient of 0.5 [17].

To demonstrate summary differences in absolute terms, we multiplied SMDs by the pooled SD of the largest study using the most common test within each domain. This allowed the summary effect to be re-expressed in terms of the original units of measurement of commonly encountered tools to show how differences may be clinically important [6].

Analyses were conducted using a combination of Microsoft Excel for Mac 2011, Stata 13.1, and Review Manager 5.3.

### Applied subgroup and sensitivity analyses

We performed a random effects meta-regression to explore potential effects of sex, number of tests, total NOS scores, and NOS components (selection, comparability, and outcome) on cognition. We used funnel plots to explore potential for publication or other biases.

## Results

### Study selection, design and characteristics

**Fig. 1:**
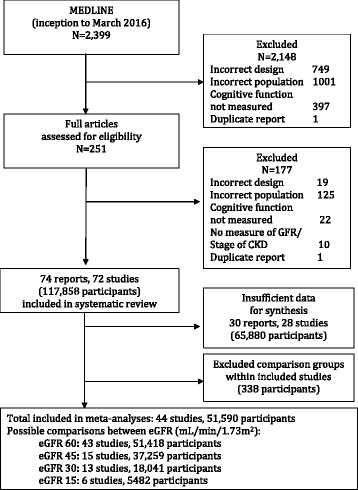
Identification and selection. PRISMA flowchart showing process of inclusion and exclusion of studies

We identified 72 eligible studies with a total of 117,858 participants (Fig. 1). Of these, 28 studies were not included in meta-analysis due to insufficient data for synthesis and inability to obtain sufficient data from authors, despite efforts. This left 44 cross-sectional and cohort studies with a total of 51,928 participants. Some studies reported data for comparator subpopulations not included in our research question (e.g. people on dialysis), so these data were excluded, leaving data on 51,590 participants for synthesis in final meta-analysis. We found no randomised trials.

Study characteristics for studies included in the meta-analysis are described in Table 1. See Additional file 2 for the complete list of studies and their characteristics that met inclusion criteria and detailed study characteristics.

**Table 1:** Characteristics of studies included in quantitative synthesis

Characteristic	Meta-analysis comparison eGFR cut point in mL/min/1.73 m^2^, studies (participants)				
	< 15	< 30	< 45	< 60	Overall
Total	6 (5482)	13 (18,041)	16 (37,259)	43 (51,418)	44 (51,575)
Region					
Americas	3 (5327)	5 (8356)	8 (17,921)	21 (27,976)	22 (28,133)
Asia & Pacific	1 (60)	4 (1728)	4 (5787)	11 (9246)	11 (9246)
Europe & Russia	2 (95)	3 (7887)	4 (13,551)	8 (13,848)	8 (13,848)
Africa	–	1 (70)	–	3 (348)	3 (348)
Study design					
Cross-sectional	6 (5482)	8 (6434)	9 (17,486)	33 (26,915)	33 (27,072)
Cohort	–	5 (11,607)	7 (19,773)	10 (24,503)	10 (24,503)
Participants^a^					
Mean age (Range), years					67.1 (18–89)
Women					52.2%
Basis of between-groups comparisons					
eGFR/proteinuria	3	11	14	31	32
CKD vs. Healthy	4	4	2	17	17
Within-CKD	1	–	–	–	1
CKD vs. other conditions	1	–	–	1	1

^a^Age and sex data are taken from whole study descriptive statistics

*CKD* chronic kidney disease, *eGFR* estimated glomerular filtration rate

The mean NOS score for cohort and cross-sectional studies was 5.8/9 and 5.4/10, respectively (see also Additional file 2 for NOS scores and demographic and health status matching for the 44 studies that were included in the meta-analysis). Although cohort studies performed well in the Selection category, cross-sectional studies tended to perform poorly. Both study types performed relatively poorly on Outcome, with very few providing sufficient detail outcome assessment procedures. Comparability under the NOS was high across most studies, however, examining demographics and disease status matching of actual CKD and comparison groups, most studies performed poorly on both. Thus, studies were primarily limited by comparison group validity when additional factors that may affect cognition were considered. People with neurological conditions (e.g. stroke) were excluded by 23 (52.3%) studies.

### Cognitive tests

Cognitive domains were measured with varying frequencies in synthesised studies (Table 2): Orientation & Attention (28 studies), Global Cognition (25), Memory (16), Executive Functions (15), Construction & Motor Praxis (11), Language (9), Concept Formation & Reasoning (6), and Perception (1). Funnel plots for Global Cognition tests showed some asymmetry, suggesting some studies showing reduced differences may be missing, or have been subject to publication bias. Funnel plots for other commonly tested cognitive domains were more symmetrical (Additional file 3).

**Table 2:** Cognitive domains of synthesised studies, including subdomains

Cognitive domain (Studies)	Sub-domains (Studies)	Description of domain	Examples
Orientation & Attention (28)	Orientation (0)	Ability to attend to one’s environment and maintain short-term or continuous focus on information or tasks	Trail Making Test, digit span, digit-symbol substitution, immediate recall
	Attention, processing speed, and working memory (28)		
Perception (1)	Visual (0)	Ability to receive and make sense of stimuli	Hooper Visual Organization, Halstead-Reitan speech sounds perception test
	Auditory (1)		
	Tactile (1)		
	Olfactory (0)		
Memory (17)	Verbal (16)	Ability to remember information, whether verbal, auditory, or visual following a period of time	California Verbal Learning Test, Wechsler Memory Scale, facial memory, visual memory, word list, long delay recall
	Visual (4)		
	Tactile (0)		
	Incidental learning (0)		
	Prospective learning (0)		
	Remote memory (0)		
Language (9)	Aphasia (3)	Ability to understand, produce, and use language appropriately	Boston Naming Test
	Verbal expression (4)		
	Verbal comprehension (3)		
	Verbal academic skills (0)		
Construction & Motor Praxis (11)	Drawing (4)	Ability to produce or orient within space 2D or 3D representations	Rey-Osterreith Complex Figure, Wechsler Adult Intelligence Scale block design and object assembly tests, Purdue Pegboard
	Assembling and building (4)		
	Motor skills (3)		
Concept Formation & Reasoning (6)	Concept formation (2)	Ability to utilise patterns and abstract concepts to solve problems	Judgment of Line Orientation, Raven’s Progressive Matrices
	Reasoning (4)		
	Mathematical procedures (0)		
Executive Functions (15)		‘Higher order’ abilities that draw on multiple other domains	Wisconsin Card Sorting Test, verbal fluency
Global Cognition (25)		Collection of domains measured in cognitive impairment (e.g. dementia and Alzheimer’s disease) screening tests; these primarily measure Orientation & Attention, Memory, and Language	MMSE, Modified MMSE (3MS), Telephone Interview for Cognitive status

### CKD patients compared to non-CKD (eGFR < 60 mL/min/1.73 m^2^ vs. ≥ 60 mL/min/1.73 m^2^)

**Fig. 2:**
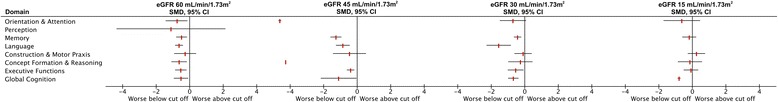
Standardised mean differences in cognitive domains within estimated glomerular filtration rate cut point groups

**Fig. 3:**
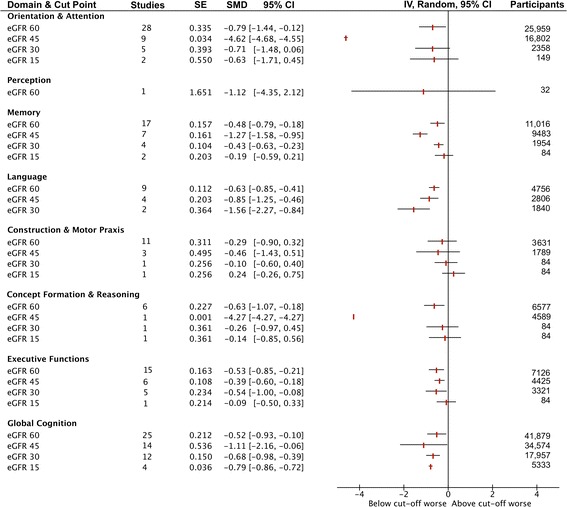
Standardised mean differences in cognitive scores comparing participants at estimated glomerular filtration rate cut points

CKD patients (eGFR < 60 mL/min/1.73 m^2^) performed more poorly than control groups (eGFR ≥ 60 mL/min/1.73 m^2^) on Orientation & Attention (SMD –0.79, 95% CI, –1.44 to –0.13), Language (SMD –0.63, 95% CI, –0.85 to –0.41), Concept Formation & Reasoning (SMD –0.63, 95% CI, –1.07 to –0.18), Executive Function (SMD –0.53, 95% CI, –0.87 to –0.21), Memory (SMD –0.48, 95% CI, –0.79 to –0.18), and Global Cognition (SMD –0.48, 95% CI, –0.72 to –0.24) tests (Figs. 2 and 3). Studies were limited primarily by comparison group validity, with very few being appropriately matched for demographics or health status.

### Stratified comparisons by CKD stage

**Fig. 4:**
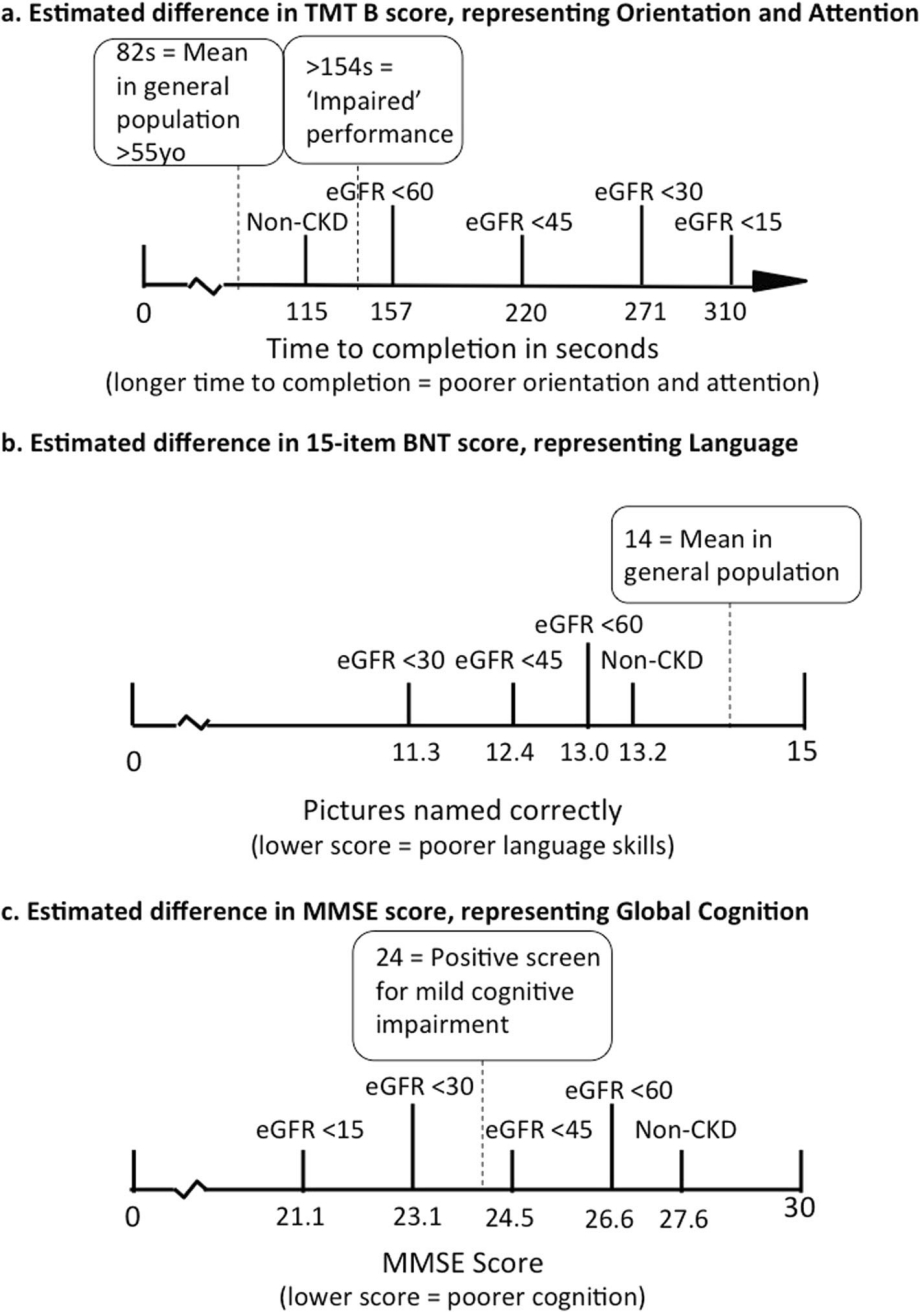
Estimated mean differences in test scores for commonly used tests

Figure 2 demonstrates changes in cognition by CKD stage, by comparing SMD above and below CKD stage eGFR cut points. Although Language scores continued to drop with lower eGFR (< 45 mL/min/1.73 m^2^, Stages 4, 3B, and non-dialysed CKD Stage 5 vs. Stage 3A and better, SMD –0.86, 95% CI, –1.25 to –46; 30 mL/min/1.73 m^2^, Stage 4 and non-dialysed Stage 5 vs. Stage 3B and better, SMD –1.56, 95% CI, –2.27 to –0.84), this was not the case for all domains (Fig. 3). Differences in Orientation & Attention were most apparent in patients with eGFR < 45 mL/min/1.73 m^2^ (Stages 4, 3B, and non-dialysed CKD Stage 5 vs. Stage 3A and better; SMD –4.62, CI –4.68 to –4.55). One large study also demonstrated that Concept Formation & Reasoning differences were most apparent at eGFR < 45 mL/min/1.73 m^2^ (Stages 4, 3B, and non-dialysed CKD Stage 5 vs. Stage 3A and better SMD –4.27, 95% CI, –4.23 to –4.27). Differences in Executive Functions were most apparent at eGFR < 30 mL/min/1.73 m^2^ (Stage 4 and non-dialysed CKD Stage 5 vs. Stage 3B and better, SMD –0.54, 95% CI, –1.00 to –0.08). Construction & Motor Praxis was unaffected by CKD at any stage (eGFR < 60 mL/min/1.73 m^2^ vs. ≥ 60 mL/min/1.73 m^2^ SMD –0.29, 95% CI, –0.90 to 0.32), and Perception (which was measured by one small study) also appeared to be unaffected (eGFR < 60 mL/min/1.73 m^2^ vs. ≥ 60 mL/min/1.73 m^2^ SMD –1.12, 95% CI, –4.35 to 2.12). Figure 4 shows estimated changes in test scores for commonly used tests in the domains of Orientation & Attention, Language, and Global Cognition.

### Applied subgroup and sensitivity analyses

**Fig. 5:**
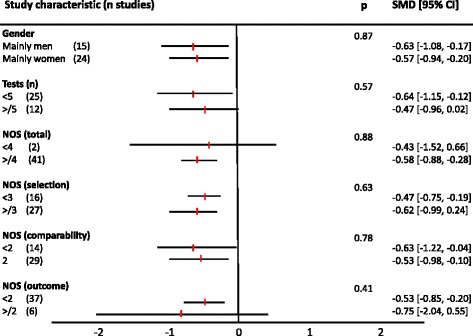
Subgroup sensitivity analyses. Subgroup sensitivity analyses showed no differences based on sex, number of cognitive tests, NOS total score, or NOS components

No differences in cognitive test scores were observed based on sex, number of tests applied, NOS total scores, or NOS component scores (Fig. 5).

## Discussion

We found cognitive changes occur early in CKD, and that these changes progress at different rates for different cognitive domains as CKD progresses and eGFR declines. This finding confirms and expands on both the trend suggested by Etgen et al.'s [1] meta-analysis that risk of cognitive impairment increases with lower eGFR and that, overall, CKD patients have poorer cognition than those without CKD. Our data, however, examined performance in individual cognitive domains rather than incidence, thus characterising the cognitive impairment. CKD patients performed worse than control groups on Orientation & Attention, Language, Concept Formation & Reasoning, Memory, Executive Function, and Global Cognition tests. The early effect of CKD on Orientation & Attention may have a knock-on effect to other cognitive domains such as Memory. This would be consistent with symptoms reported by patients, such as a general cognitive ‘slowness’ or ‘haziness’ that may be associated with word finding difficulties or trouble following logical arguments. Each domain followed a unique pattern of decline, with some such as language continuing to decline and others plateauing or only becoming problematic at a certain point. As this review excluded studies of AKI patients, it would be inappropriate to draw conclusions about the trajectory of AKI patients, including AKI in the context of CKD. The trajectory of CKD-related cognitive impairment does not appear to manifest as a rapid decline, as demonstrated by shifts across CKD stages which typically occur over years. Such a clinical scenario involving rapid deterioration in cognitive function would necessitate evaluation for delirium, sepsis, AKI, stroke, traumatic brain injury, and other clinically indicated differential diagnoses.

### Applicability of findings in clinical practice

Although there may be overlap in some of the deficits in severe CKD-related cognitive impairment, the pattern of cognitive impairment evident in CKD patients does not follow that of any known dementia syndrome nor traumatic brain injury. There is thus a lack of fit with existing services designed for cognitive impairment in adults and a lack of guidelines for addressing CKD patients’ cognition-related needs. Given the potential for cognitive impairment, CKD patients may need assistance navigating care pathways, weighing up treatment options, compiling advice from multiple sources, maintaining medication regimens, and remembering appointments and pharmacy pick ups. Thus, early liaison with multidisciplinary nephrology and community services to support patient independence and coordination with all providers may be an important step in the care of patients as they navigate the progression of their CKD.

Even as eGFR fell below 60 mL/min/1.73 m^2^, using our cross-sectional meta-analytic design, early impairment was seen in the areas of Orientation & Attention, Language, Concept Formation & Reasoning, Memory, and Executive Functions. Differences in mean scores could also be detected on Global Cognition screening tests. Although scores on individual tests may not, at this point, fall below standardised cut off points for cognitive impairment, it may be important for clinicians to track patient cognition over time from baseline and for patients to know that changes they experience are probably due to kidney disease rather than age or mental illness.

As cognitive impairment can be severe by CKD stages 4 and 5, a patient’s capacity to make healthcare decisions can be greatly diminished [12]. Health literacy refers to “*the wide range of skills, and competencies that people develop to seek out, comprehend, evaluate and use health information and concepts to make informed choices, reduce health risks and increase quality of life*” [18]. It is one aspect of health decision-making, and although low literacy is associated with low health literacy, the two are not interchangeable [19]. Even before capacity is diminished, basic health literacy may be affected through mechanisms such as poor attention and memory as well as reduced language and reasoning skills. The link between poor health literacy and outcomes is well established, and clinicians can work to support health literacy in CKD patients to improve quality of care [20–24]. There is a clear need for resources that accommodate CKD-related cognitive impairment, and knowledge measures have been shown useful in other populations to gauge usefulness of such aids [25]. By screening CKD patients for impaired Orientation & Attention using tools such as the Trail Making Test B, early identification of difficulties may lead to targeted interventions and early discussions with the patient and their family.

### Strengths and limitations of the current study

A strength of our work is the pragmatic and systematic approach to summarising cognitive impairment in CKD from a broad reaching neuropsychological perspective. It is important to acknowledge that we have synthesised the average effects that have been observed and that the cognitive trajectory of an individual may deviate from the average effect. Similarly, where we have shown a distinct pattern of cognitive change associated with CKD, dementia is common in older patients and thus may coexist within an individual. It should be noted that these patterns were identified through the analysis of cross-sectional data and not longitudinal data. A given person may not exhibit a particular pattern of cognitive impairment, but we see that, for a given eGFR in a person with CKD, certain deficits may be expected.

Although most studies controlled for at least two demographic variables (age, education and sex were common), many factors are associated with differences in cognitive testing scores. Studies were limited primarily by comparison group validity, with very few being well matched for demographics and health status. As a result, the degree of cognitive impairment in CKD patients may be biased. Consensus on a standardised test battery, given the patterns of deficit we have observed, would make comparison of studies and subgroups clearer and has recently been identified as a research priority [26]. Future research should aim to test well matched CKD and control groups across a range of cognitive domains and compare stages of CKD. Additionally, more research is needed examining the domain of Perception in CKD to provide more definitive results.

## Conclusions

CKD affects several cognitive domains but not predominantly those associated with dementia syndromes. Domains particularly affected are Orientation & Attention and Concept Formation & Reasoning, while other domains affected include Memory, Language, and Executive Functions. Global Cognition measures appear to be useful in screening for CKD-related cognitive impairment but may not correlate well with renal function. More research comparing CKD patients with well matched non-CKD patients would improve understanding of CKD-related cognitive impairment.

## Additional files

Additional file 1: Medline search strategy. (PDF 61.2 kb)
Additional file 2: Characteristics of studies. (PDF 224 kb)
Additional file 3: Funnel plots. (PDF 259 kb)
Additional file 4: Meta-analysis for eGFR 60. (XLSX 77.4 kb)
Additional file 5: Meta-analysis for eGFR 45. (XLSX 52.4 kb)
Additional file 6: Meta-analysis for eGFR 30. (XLSX 47.5 kb)
Additional file 7: Meta-analysis for eGFR 15. (XLSX 37.2 kb)

## Abbreviations

AKI: acute kidney injury
CKD: chronic kidney disease
eGFR: estimated glomerular filtration rate
MMSE: Mini Mental State Examination
NOS: Newcastle Ottawa Scale
SMD: standardised mean difference
