# Supplementary material for: Cognition in chronic kidney disease: a systematic review and meta-analysis
Source: BMC Med. 2016 Dec 14;14:206. doi: 10.1186/s12916-016-0745-9 (PMC5155375; doi:10.1186/s12916-016-0745-9)
Supplement: Additional file 1: — Medline search strategy. (PDF 61.2 kb) [file 12916_2016_745_MOESM1_ESM.pdf]

Title: Cognition in chronic kidney disease: a systematic review and meta-analysis

Authors: I Berger, S Wu, P Masson, PJ Kelly, FA Duthie, W Whiteley, D Parker, D Gillespie, AC Webster

Additional File 1. Medline Search Strategy

1. Cognition disorders/
2. Mild Cognitive Impairment/
3. Dementia/
4. exp Aphasia, Primary Progressive/
5. exp Dementia, Vascular/
6. Diffuse Neurofibrillary Tangles with Calcification/
7. exp Frontotemporal Lobar Degeneration/
8. (cognit* adj3 (impair* or disorder* or def*)).tw.
9. dement*.tw.
10. aphasi*.tw.
11. (fronto?temporal lob* or fronto?temporal dement*).tw.
12. diffuse neuro?fibrillary tangles with calcification.tw.
13. or/1-12
14. exp Renal Replacement Therapy/
15. h?emo?dialysis.tw.
16. h?emo?filtration.tw.
17. h?emo?diafiltration.tw.
18. dialysis.tw.
19. (CAPD or CCPD or APD).tw.
20. (kidney transplant* or renal transplant* or kidney graft* or renal graft*).tw.
21. Renal Insufficiency/
22. exp Renal Insufficiency, Chronic/
23. exp Kidney Diseases/
24. Uremia/
25. (kidney disease* or renal disease* or kidney failure or renal failure).tw.
26. (ESRF or ESKF or ESRD or ESKD).tw.
27. (CKF or CKD or CRF or CRD).tw.
28. (predialysis or pre-dialysis).tw.
29. ur?emi$.tw.
30. or/14-29
31. and/13,30
32. exp Neuropsychological Tests/
33. Language Tests/
34. mini mental state exam*.tw.
35. frontal assessment battery.tw.
36. (wechsler adj3 (intelligen* or memory)).tw.
37. (span adj2 (digit or spatial or symbol)).tw.
38. matrix reasoning.tw.
39. (clock drawing adj2 (test or task)).tw.
40. halstead reitan battery.tw.
41. trail making test.tw.
42. tactual performance test.tw.
43. block design test.tw.
44. letter number sequencing test.tw.
45. finger tapping test.tw.
46. (reitan adj4 (test or task or exam*)).tw.

47. token test.tw.
48. boston naming test.tw.
49. stroop test.tw.
50. (word adj2 (rec* or list or associat* or context) adj2 (test or task or exam*)).tw.
51. (picture adj2 (completion or recognition or presentation)).tw.
52. (fluency adj2 (verbal or design or animal or lexical) adj3 (test or task or exam*)).tw.
53. (memory adj3 (spatial or numeric or working) adj3 (test or task or exam*)).tw.
54. stockings of cambridge.tw.
55. rey osterrieth complex figure test.tw.
56. buschke selective reminding test.tw.
57. wisconsin card sorting test.tw.
58. progressive matri*.tw.
59. cognitive assessment screening.tw.
60. ((simple or choice) adj3 reaction adj3 (speed or time)).tw.
61. progressive matri*.tw.
62. hooper visual.tw.
63. "visual object and space perception battery".tw.
64. (neuropsychiatric adj (inventory or battery)).tw.
65. neuropsychological test*.tw.
66. (3MS or PASAT or WASI or WAIS).tw.
67. or/32-66
68. and/31,67
69. executive function/
70. memory/
71. language/
72. attention/
73. memory, long-term/
74. memory, short-term/
75. memory, episodic/
76. visual perception/
77. space perception/
78. psychomotor performance/
79. motor skills/
80. "task performance and analysis"/
81. global cognitive function*.tw.
82. executive function*.tw.
83. (memory adj3 (disorder* or function* or def*)).tw.
84. (language adj3 (function* or disorder* or def*)).tw.
85. ((attention or concentration) adj3 (def* or disorder* or function*)).tw.
86. ((visual or spatial or visualspatial or visuospatial) adj3 (function* or abilit* or awareness)).tw.
87. (mental adj3 (efficien* or automatation or fluency) adj3 (def* or disorder* or function*)).tw.
88. intelligence/
89. (intelligence adj3 (quotient or def* or abilit*)).tw.
90. ((motor or psychomotor) adj3 (abilit* or function* or def*)).tw.

Title: Cognition in chronic kidney disease: a systematic review and meta-analysis

Authors: I Berger, S Wu, P Masson, PJ Kelly, FA Duthie, W Whiteley, D Parker, D Gillespie, AC Webster

91. or/69-90
92. and/30,91
93. or/31,68,92
94. remove duplicates from 93
95. limit 94 to humans
96. from 95 keep 1-1000
97. from 95 keep 1001-2000
98. from 95 keep 2001-2296

Title: Cognition in chronic kidney disease: a systematic review and meta-analysis

Authors: I Berger, S Wu, P Masson, PJ Kelly, FA Duthie, W Whiteley, D Parker, D Gillespie, AC Webster
