# Supplementary material for: Cognition in chronic kidney disease: a systematic review and meta-analysis
Source: BMC Med. 2016 Dec 14;14:206. doi: 10.1186/s12916-016-0745-9 (PMC5155375; doi:10.1186/s12916-016-0745-9)
Supplement: Additional file 2: — Characteristics of studies. (PDF 224 kb) [file 12916_2016_745_MOESM2_ESM.pdf]

**Title:** Cognition in chronic kidney disease: a systematic review and meta-analysis

**Authors:** I Berger, S Wu, P Masson, PJ Kelly, FA Duthie, W Whiteley, D Parker, D Gillespie, AC Webster

**Additional File 2. Characteristics of Studies**

Table 1. Characteristics of Studies Included in Meta-Analysis

| Reference | Country | Included N | Cognitive domains investigated* | Basis of between-groups comparisons | Newcastle Ottawa scale score | | | | Comparison groups well matched for | |
|---|---|---|---|---|---|---|---|---|---|---|
| | | | | | Selection (/5)† | Compar-ability (/2) | Outcome (/3) | Total (/10) † | Demo-graphics | Disease State |
| ***Studies contributing to <60 mL/min/1.73m²*** | | | | | | | | | | |
| Apprecida de Lima Conde 2010 [1] | Brazil | 62 | 1,5 | CKD diagnosis | 2 | 2 | 1 | 5 | ✓ | |
| Caltagirone 1987 [2] | Italy | 43 | 1,3,4,5,6,7 | CKD diagnosis | 2 | 2 | 1 | 5 | | |
| Etgen 2009 [3] ‡ | Germany | 3679 | 8 | creatinine clearance | 3 | 2 | 2 | 7 | | |
| Feng 2012 [4] ‡ | Singapore | 1315 | 8 | eGFR | 4 | 1 | 1 | 6 | | |
| Gelb 2008 [5] | Canada | 94 | 1,3 | 'healthy', eGFR | 3 | 0 | 1 | 4 | | |
| Hailpern 2007 [6] | USA | 4849 | 1 | eGFR | 4 | 2 | 3 | 9 | | |
| Hart 1983 [7] | USA | 38 | 1,3,5 | CKD diagnosis | 2 | 2 | 1 | 5 | | ✓ |
| Heidbreder 1979 [8] | Germany | 65 | 1 | 'normal', mild hypertension without renal disease, CKD diagnosis | 2 | 1 | 1 | 4 | | |
| Helmer 2011 [9] ‡ | France | 7806 | 8 | eGFR | 3 | 0 | 1 | 4 | | |
| Helmes 2015 [10] | Canada | 1571 | 1,4,5,6,7,8 | CKD diagnosis | 3 | 2 | 1 | 6 | | |
| Jassal 2010 [11] ‡ | USA | 1346 | 1,7 | albumin-creatinine ratio | 3 | 2 | 1 | 6 | | |
| Kato 2012 [12] | Japan | 43 | 8 | CKD diagnosis | 2 | 1 | 1 | 4 | | |
| Khatri 2009 [13] ‡ | USA | 2172 | 8 | eGFR | 3 | 2 | 1 | 6 | | |
| Kurella 2005 [14] ‡ | USA | 1015 | 1,3,4,7,8 | eGFR | 2 | 2 | 0 | 4 | | |
| Kurella 2005 [15] | USA | 3034 | 8 | eGFR | 4 | 2 | 1 | 7 | | |
| Kuriyama 2013 [16] ‡ | Japan | 273 | 7,8 | eGFR | 4 | 2 | 3 | 9 | | |
| Lee 2011 [17] | Korea | 529 | 1,3,4,5,7,8 | eGFR | 3 | 2 | 1 | 6 | | |

**Title:** Cognition in chronic kidney disease: a systematic review and meta-analysis

**Authors:** I Berger, S Wu, P Masson, PJ Kelly, FA Duthie, W Whiteley, D Parker, D Gillespie, AC Webster

| | | | | | | | | | | |
|---|---|---|---|---|---|---|---|---|---|---|
| Lee 2015 [18] | Japan | 4686 | 1,3,6,8 | eGFR | 3 | 0 | 1 | 4 | | |
| Madan 2007 [19] | India | 45 | 8 | ‘healthy’, eGFR | 2 | 2 | 1 | 5 | | |
| Murea 2015 [20] | USA | 478 | 1,3,8 | eGFR | 3 | 1 | 1 | 5 | | ✓ |
| Nasser 2012 [21] | Egypt | 70 | 1,8 | ‘healthy’, eGFR | 3 | 2 | 1 | 6 | | |
| Ogunrin 2006 [22] | Nigeria | 120 | 1,3 | ‘healthy’, eGFR | 3 | 2 | 1 | 6 | | |
| Raphael 2012 [23] | USA | 5086 | 8 | CKD diagnosis | 3 | 2 | 3 | 8 | | |
| Rogova 2013 [24] | Russia | 51 | 1,3,7,8 | eGFR | 2 | 0 | 1 | 3 | | |
| Ryan 1980 [25] | USA | 48 | 4,5,7 | CKD diagnosis | 2 | 2 | 1 | 5 | | |
| Ryan 1981 [26] | USA | 32 | 1,2,4,5,6,7 | CKD diagnosis | 2 | 2 | 1 | 5 | | |
| Sanchez-Roman 2011 [27] | Mexico | 84 | 1,3,5,6,7 | eGFR | 2 | 2 | 1 | 5 | ✓ | |
| Seidel 2014 [28] | Germany | 138 | 1,5,7 | eGFR | 2 | 2 | 3 | 7 | ✓ | |
| Sink 2015 [29] | USA | 263 | 1,3,8 | eGFR | 3 | 2 | 1 | 6 | | |
| Silverwood 2014 [30] | UK | 2036 | 1,3 | eGFR | 4 | 2 | 1 | 7 | | |
| Slinin 2008 [31] ‡ | USA | 5529 | 1,8 | eGFR | 2 | 2 | 1 | 5 | | |
| Spencer 2013 [32] | USA | 258 | 6 | CKD diagnosis | 2 | 1 | 1 | 4 | | |
| Sujic 1997 [33] | Serbia | 30 | 3,8 | CKD diagnosis | 2 | 2 | 1 | 5 | | |
| Szerlip 2015 [34] | USA | 437 | 1,3,4,5,7,8 | eGFR | 3 | 1 | 1 | 5 | | |
| Thornton 2007 [35] | Canada | 106 | 1,3 | ‘healthy’, eGFR | 3 | 2 | 1 | 6 | | |
| Tsai 2010 [36] | Taiwan | 256 | 1,3,4,7 | eGFR | 3 | 2 | 1 | 6 | | |
| Wang 2010 [37] ‡ | China | 1243 | 8 | eGFR | 4 | 1 | 2 | 7 | | |
| Weng 2012 [38] ‡ | Taiwan | 125 | 8 | eGFR | 3 | 0 | 1 | 4 | | |
| Williams 2013 [39] | Nigeria | 158 | 1 | ‘healthy’, eGFR | 3 | 2 | 1 | 6 | | |
| Yaffe 2010 [40] | USA | 825 | 1,3,4,7,8 | eGFR | 3 | 2 | 1 | 6 | | |
| Yang 2010 [41] | Taiwan | 228 | 8 | eGFR | 2 | 0 | 0 | 2 | | |
| Yao 2011 [42] | Japan | 503 | 1 | eGFR | 3 | 2 | 1 | 6 | | |
| Zammit 2015 [43] § | USA | 649 | 1,5 | eGFR | 3 | 1 | 1 | 5 | | ✓ |
| ***Studies contributing to <45 mL/min/1.73m$^2$*** | | | | | | | | | | |
| Etgen 2009 [3] ‡ | Germany | 3679 | 8 | creatinine | 3 | 2 | 2 | 7 | | |

| | | | | | | | | | | |
|---|---|---|---|---|---|---|---|---|---|---|
| | | | | clearance | | | | | | |
| Helmer 2011 [9] ‡ | France | 7806 | 8 | eGFR | 3 | 0 | 1 | 4 | | |
| Jassal 2010 [11] ‡ | USA | 1346 | 1,7 | albumin-creatinine ratio | 3 | 2 | 1 | 6 | | |
| Kurella 2005 [14] ‡ | USA | 1015 | 1,3,4,7,8 | eGFR | 2 | 2 | 0 | 4 | | |
| Kurella 2005 [15] | USA | 3034 | 8 | eGFR | 4 | 2 | 1 | 7 | | |
| Kuriyama 2013 [16] ‡ | Japan | 273 | 7,8 | eGFR | 4 | 2 | 3 | 9 | | |
| Lee 2011 [17] | Korea | 703 | 1,3,4,5,7,8 | eGFR | 3 | 2 | 1 | 6 | | |
| Lee 2015 [18] | Japan | 4686 | 1,3,6,8 | eGFR | 3 | 0 | 1 | 4 | | |
| Raphael 2012 [23] | USA | 5086 | 8 | CKD diagnosis | 3 | 2 | 3 | 8 | | |
| Silverwood 2014 [30] | UK | 2036 | 1,3 | eGFR | 4 | 2 | 1 | 7 | | |
| Slinin 2008 [31] ‡ | USA | 5529 | 1,8 | eGFR | 2 | 2 | 1 | 5 | | |
| Sujic 1997 [33] | Serbia | 30 | 3,8 | CKD diagnosis | 2 | 2 | 1 | 5 | | |
| Szerlip 2015 [34] | USA | 437 | 1,3,4,5,7,8 | eGFR | 3 | 1 | 1 | 5 | | |
| Weng 2012 [38] ‡ | Taiwan | 125 | 8 | eGFR | 3 | 0 | 1 | 4 | | |
| Yaffe 2010 [40] | USA | 825 | 1,3,4,7,8 | eGFR | 3 | 2 | 1 | 6 | | |
| Zammit 2015 [43] § | USA | 649 | 1,5 | eGFR | 3 | 1 | 1 | 5 | | ✓ |
| ***Studies contributing to <30 mL/min/1.73m$^2$*** | | | | | | | | | | |
| Feng 2012 [4] ‡ | Singapore | 1315 | 8 | eGFR | 4 | 1 | 1 | 6 | | |
| Helmer 2011 [9] ‡ | France | 7806 | 8 | eGFR | 3 | 0 | 1 | 4 | | |
| Jassal 2010 [11] ‡ | USA | 1346 | 1,7 | albumin-creatinine ratio | 3 | 2 | 1 | 6 | | |
| Kurella 2005 [14] ‡ | USA | 1015 | 1,3,4,7,8 | eGFR | 2 | 2 | 0 | 4 | | |
| Madan 2007 [19] | India | 60 | 8 | ‘healthy’, eGFR | 2 | 2 | 1 | 5 | | |
| Nasser 2012 [21] | Egypt | 70 | 1,8 | ‘healthy’, eGFR | 3 | 2 | 1 | 6 | | |
| Raphael 2012 [23] | USA | 5086 | 8 | CKD diagnosis | 3 | 2 | 3 | 8 | | |
| Rogova 2013 [24] | Russia | 51 | 1,3,7,8 | eGFR | 2 | 0 | 1 | 3 | | |
| Sanchez-Roman 2011 [27] | Mexico | 84 | 1,3,5,6,7 | eGFR | 2 | 2 | 1 | 5 | ✓ | |
| Sujic 1997 [33] | Serbia | 30 | 3,8 | CKD diagnosis | 2 | 2 | 1 | 5 | | |
| Weng 2012 [38] ‡ | Taiwan | 125 | 8 | eGFR | 3 | 0 | 1 | 4 | | |
| Yaffe 2010 [40] | USA | 825 | 1,3,4,7,8 | eGFR | 3 | 2 | 1 | 6 | | |

**Title:** Cognition in chronic kidney disease: a systematic review and meta-analysis

**Authors:** I Berger, S Wu, P Masson, PJ Kelly, FA Duthie, W Whiteley, D Parker, D Gillespie, AC Webster

| | | | | | | | | | | |
|---|---|---|---|---|---|---|---|---|---|---|
| Yang 2010 [41] | Taiwan | 228 | 8 | eGFR | 2 | 0 | 0 | 2 | | |
| ***Studies contributing to <15 mL/min/1.73m²*** | | | | | | | | | | |
| Heidbreder 1979 [8] | Germany | 65 | 1 | 'normal', mild hypertension without renal disease, CKD diagnosis | 2 | 1 | 1 | 4 | | |
| Kurella 2004 [44] | USA | 157 | 8 | eGFR (CKD only) | 2 | 2 | 1 | 5 | | ✓ |
| Madan 2007 [19] | India | 60 | 8 | 'healthy', eGFR | 2 | 2 | 1 | 5 | | |
| Raphael 2012 [23] | USA | 5086 | 8 | CKD diagnosis | 3 | 2 | 3 | 8 | | |
| Sanchez-Roman 2011 [27] | Mexico | 84 | 1,3,5,6,7 | eGFR | 2 | 2 | 1 | 5 | ✓ | |
| Sujic 1997 [33] | Serbia | 30 | 3,8 | CKD diagnosis | 2 | 2 | 1 | 5 | | |

* 1, Orientation & Attention; 2, Perception; 3, Memory; 4, Language; 5, Construction & Motor Praxis; 6, Concept Formation & Reasoning; 7, Executive Functions; 8, Global Cognition.
† Except for cohort/longitudinal studies, which are out of 4 for selection, resulting in a possible total score of 9.
‡ Cohort/longitudinal study. All other studies are cross-sectional.
§ Zammit (2015) is a cohort study, but only baseline data were presented in the cited article.

**Title:** Cognition in chronic kidney disease: a systematic review and meta-analysis

**Authors:** I Berger, S Wu, P Masson, PJ Kelly, FA Duthie, W Whiteley, D Parker, D Gillespie, AC Webster

Table 2. Characteristics of Included Studies with Insufficient Data for Synthesis

| Reference | Country | Total participants | Cognitive domains investigated* |
|---|---|---|---|
| Afsar 2011 [45] | Turkey | 247 | 8 |
| Afsar 2013 [46] | Turkey | 109 | 8 |
| Arismendi-Morillo 2010 [47] | Venezuela | 4 | 5 |
| Balasubramaniam 1972 [48] | UK | 27 | 6 |
| Barzilay 2011 [49] † | Austria, Belgium, Czech Republic, Germany, Greece, Netherlands, Finland, Norway, Sweden, South Africa, United Arab Emirates, South Korea | 28,384 | 8 |
| Buchman 2009 [50] † | USA | 886 | 1,3,4,6,7,8 |
| Corrao 2015 [51] | Italy | 1384 | 8 |
| Egbi 2015 [52] | Nigeria | 190 | 8 |
| Elias 2009 [53] | USA | 923 | 1,2,3,4,5,6,7 |
| Giallauria 2015 [54] | Italy | 2281 | 8 |
| Ginn 1975 [55] | USA | Not specified | 1,3,6 |
| Jassal 2008 [56] | Canada | 99 | 1,3,6 |
| Kang 2012 [57] | USA | 169 | 1,3,7,8 |
| Kurella 2004 [58] | USA | 160 | 1,3,8 |
| Kurella Tamura 2008 [59] | USA | 23,405 | 8 |
| Kurella Tamura 2011 [60] † | USA | 19,399 | 8 |
| Kurella Tamura 2011 [61] | USA | 3591 | 8 |
| Marchesi 1978 [62] | Italy | 29 | 3,5,6,7 |
| Morosanu 2011 [63] | Romania | 63 | 8 |
| Nulsen 2008 [64] | UK | 132 | 8 |
| Post 2010 [65] | USA | 51 | 1,3,4,7,8 |
| Rabinowitz 1980 [66] | South Africa | 31 | 6 |
| Romjin 2015 [67] | The Netherlands | 581 | 8 |
| Sajjad 2010 [68] † | USA | 1764 | 1,3,7,8 |
| Srivanitchapoom 2013 [69] | Thailand | 317 | 8 |
| Takuma 1987 [70] † | Japan | 62 | 1 |
| Terawaki 2008 [71] | Japan | 26 | 7,8 |
| Teschan 1979 [72] † | USA | 177 | 1 |
| Tryc 2011 [73] | Germany | 78 | 1,3,5 |

**Title:** Cognition in chronic kidney disease: a systematic review and meta-analysis

**Authors:** I Berger, S Wu, P Masson, PJ Kelly, FA Duthie, W Whiteley, D Parker, D Gillespie, AC Webster

| | | | |
|---|---|---|---|
| Williams 2013 [74] | Nigeria | 158 | 1,8 |
| Yamamoto 2011 [75] | Japan | 224 | 8 |

* 1, Orientation & Attention; 2, Perception; 3, Memory; 4, Language; 5, Construction & Motor Praxis; 6, Concept Formation & Reasoning; 7, Executive Functions; 8, Global Cognition.

† Cohort/longitudinal study. All other studies are cross-sectional.

**Title:** Cognition in chronic kidney disease: a systematic review and meta-analysis

**Authors:** I Berger, S Wu, P Masson, PJ Kelly, FA Duthie, W Whiteley, D Parker, D Gillespie, AC Webster

## References

1. Apprecida de Lima Conde S, Fernandes N, Rossi dos Santos F, Chouab A, Peruzzi da Mota MME, Bastos, MG. Cognitive decline, depression and quality of life in patients at different stages of chronic kidney disease. J Bras Nefrol. 2010;32(3):242-8.
2. Caltagirone C, Carlesimo A, Meloni C, Morosetti M, Taccone-Gallucci M, Vicari S, Casciani CU. Valutazione delle funzioni cognitive in soggetti con insufficienza renale cronica [Evaluation of cognitive functions in patients with chronic renal insufficiency]. Riv Neurol, 1987. 57(5):308-12.
3. Etgen T, Sander D, Chonchol M, Briesenick C, Poppert H, Forstl H, Bickel H. Chronic kidney disease is associated with incident cognitive impairment in the elderly: the INVADE study. Nephrol Dial Transplant. 2009;24(10):3144-50.
4. Feng L, Yap KB, Yeoh LY, Ng TP. Kidney function and cognitive and functional decline in elderly adults: findings from the Singapore longitudinal aging study. J Am Geriatr Soc. 2012;60(7):1208-14.
5. Gelb S, Shapira RJ, Hill A, Thornton WL. Cognitive outcome following kidney transplantation. Nephrol Dial Transplant. 2008;23(3):1032-8.
6. Hailpern SM, Melamed ML, Cohen HW, Hostetter TH. Moderate chronic kidney disease and cognitive function in adults 20 to 59 years of age: Third National Health and Nutrition Examination Survey (NHANES III). J Am Soc Nephrol. 2007;18(7):2205-13.
7. Hart RP, Pederson JA, Czerwinski AW, Adams RL. Chronic renal failure, dialysis, and neuropsychological function. J Clin Neuropsychol. 1983;5(4):301-12.
8. Heidbreder E, Pagel G, Heidland A. Vigilitätsstörungen bei chronischer Niereninsuffizienz [Vigilance of patients with chronic renal insufficiency]. Med Klin. 1979. 74(49):1861-66.
9. Helmer C, Stengel B, Metzger M, Froissart M, Massy ZA, Tzourio C, Berr C, Dartigues JF. Chronic kidney disease, cognitive decline, and incident dementia: the 3C Study. Neurol. 2011;77(23):2043-51.
10. Helmes E, Østbye T, Steenhuis R. Is there a multiplicative adverse effect of cardiovascular and kidney disease on neuropsychological measures? Appl Neuropsychol Adult. 2015;22(3):209-14.
11. Jassal SK, Kritz-Silverstein D, Barrett-Connor E. A prospective study of albuminuria and cognitive function in older adults: the Rancho Bernardo study. Am J Epidemiol. 2010;171(3): 277-86.
12. Kato M, Kawaguchi K, Nakai S, Murakami K, Hori H, Ohashi A, et al. Potential therapeutic system for Alzheimer's disease: removal of blood ABs by hemodialyzers and its effect on the cognitive functions of renal-failure patients. J Neural Transm. 2012;119(12):1533-44.
13. Khatri M, Nickolas T, Moon YP, Paik MC, Rundek T, Elkind MSV, et al. CKD associates with cognitive decline. J Am Soc Nephrol. 2009;20(11):2427-32.
14. Kurella M, Yaffe K, Shlipak MG, Wenger NK, Chertow GM. Chronic kidney disease and cognitive impairment in menopausal women. Am J Kidney Dis. 2005;45(1): 66-76.
15. Kurella M, Chertow, GM, Fried LF, Cummings SR, Harris T, Simonsick E, et al. Chronic kidney disease and cognitive impairment in the elderly: the health, aging, and body composition study. J Am Soc Nephrol. 2005;16(7):2127-33.
16. Kuriyama N, Mizuno T, Ohshima Y, Yamada K, Ozaki E, Shigeta M, et al. Intracranial deep white matter lesions (DWLs) are associated with chronic kidney disease (CKD) and cognitive impairment: a 5-year follow-up magnetic resonance imaging (MRI) study. Arch Gerontol Geriatr. 2013;56(1):55-60.
17. Lee JJ, Chin HJ, Byun M-S, Choe JY, Park JH, Lee SB, et al. Impaired frontal executive function and predialytic chronic kidney disease. J Am Geriatr Soc. 2011;59(9):1628-35.
18. Lee SC, Shimada H, Park H, Makizako H, Lee S, Doi T, et al. The association between kidney function and cognitive decline in community-

dwelling, elderly Japanese people. J Am Med Dir Assoc. 2015;16(4):349.e341-5.
19. Madan P, Kalra OP, Agarwal S, Tandon OP. Cognitive impairment in chronic kidney disease. Nephrol Dial Transplant. 2007;22(2): 440-4.
20. Murea M, Hsu F-C, Cox AJ, Hugenschmidt CH, Xu J, Adams JN, et al. Structural and functional assessment of the brain in European Americans with mild-to-moderate kidney disease: Diabetes Heart Study-MIND. Nephrol Dial Transplant. 2015;30(8):1322-9.
21. Nasser MET, Shwki S, El Shahawy Y, Sany D. Assessment of cognitive dysfunction in kidney disease. Saudi J Kidney Dis Transplant. 2012;23(6): 1208-14.
22. Ogunrin AO, Unuigbe EI, Azubuike C. Memory and perceptuo-motor performance in Nigerians with chronic renal impairment. Med Sci Monit. 2006;12(12): CR535-539.
23. Raphael KL, Wei G, Greene T, Baird BC, Beddhu S. Cognitive function and the risk of death in chronic kidney disease. Am J Nephrol. 2012;35(1): 49-57.
24. Rogova IV, Fomin VV, Damulin IV, Shashkova EV. Specific features of cognitive impairments in patients with predialysis chronic kidney disease. Ter Arkh. 2013;85(6):25-30.
25. Ryan JJ, Souheaver GT, DeWolfe AS. Intellectual deficit in chronic renal failure. A comparison with neurological and medical-psychiatric patients. J Nervous Mental Dis. 1980;168(12): 763-7.
26. Ryan JJ, Souheaver GT, DeWolfe AS. Halstead-Reitan Test results in chronic hemodialysis. J Nervous Mental Dis. 1981;169(5):311-4.
27. Sánchez-Román S, Ostrosky-Solis F, Morales-Buenrostro LE, Nogués-Vizcaíno MG, Alberu J, McClintock SM. Neurocognitive profile of an adult sample with chronic kidney disease. J Int Neuropsychol Soc. 2011;17(1):80-90.
28. Seidel UK, Gronewold J, Volsek M, Todica O, Kribben A, Bruck H, et al. The prevalence, severity, and association with HbA1c and fibrinogen of cognitive impairment in chronic kidney disease. Kidney Int. 2014:693-702.
29. Sink KM, Divers J, Whitlow CT, Palmer ND, Smith SC, Xu J, et al. Cerebral structural changes in diabetic kidney disease: African American-Diabetes Heart Study MIND. Diabetes Care. 2015;38(2):206-12.
30. Silverwood RJ, Richards M, Pierce M, Hardy R, Sattar N, Ferro C, et al. Cognitive and kidney function: results from a British birth cohort reaching retirement age. PLoS One. 2014;9(1): e86743.
31. Slinin Y, Paudel ML, Ishani A, Taylor BC, Yaffe K, Murray AM, et al. Kidney function and cognitive performance and decline in older men. J Am Geriatr Soc. 2008;56(11): 2082-8.
32. Spencer RJ, Wendell CR, Giggey PP, Seliger SL, Katzel LI, Waldstein SR. Judgment of Line Orientation: an examination of eight short forms. J Clin Exp Neuropsychol. 2013;35(2):160-6.
33. Sujic R, Vukovic M. Kognitivni poremećaji kod bolesnika sa hroničnom bubrežnom insuficijencijom [Cognitive disorders in patients with chronic renal insufficiency]. Vojnosanit Pregl. 1997;54(6):555-63.
34. Szerlip HM, Edwards ML, Williams BJ, Johnson LA, Vintimilla RM, O'Bryant SE. Association between cognitive Impairment and chronic kidney disease in Mexican Americans. J Am Geriatr Soc. 2015;63(10):2023-8.
35. Thornton WL, Shapiro RJ, Deria S, Gelb S, Hill A. Differential impact of age on verbal memory and executive functioning in chronic kidney disease. J Int Neuropsychol Soc. 2007;13(2):344-53.
36. Tsai C-F, Wang S-J, Fuh J-L. Moderate chronic kidney disease is associated with reduced cognitive performance in midlife women. Kidney Int, 2010;78(6):605-10.
37. Wang F, Zhang L, Liu L, Wang H. Level of kidney function correlates with cognitive decline. Am J Nephrol. 2010;32(2):117-21.

**Title:** Cognition in chronic kidney disease: a systematic review and meta-analysis

**Authors:** I Berger, S Wu, P Masson, PJ Kelly, FA Duthie, W Whiteley, D Parker, D Gillespie, AC Webster


38. Weng S-C, Shu K-H, Tang Y-J, Sheu, WH-H, Tarng D-C, Wu, M-J, et al. Progression of cognitive dysfunction in elderly chronic kidney disease patients in a veteran's institution in central Taiwan: a 3-year longitudinal study. Intern Med. 2012;51(1):29-35.
39. Williams UE, Owolabi MO, Ogunniyi A, Ezunu EO. Prevalence and pattern of neurocognitive impairment in Nigerians with stages 3 to 5 chronic kidney disease. ISRN Neurol. 2013. doi: 10.1155/2013/374890
40. Yaffe K, Ackerson L, Kurella Tamura M, Le Blanc P, Kusek JW, Sehgal AR, et al. Chronic kidney disease and cognitive function in older adults: findings from the chronic renal insufficiency cohort cognitive study. J Am Geriatr Soc. 2010;58(2):338-45.
41. Yang AC, Tsai S-J, Yeh H-L, Chen J-Y, Liou Y-J, Hwang J-P, Hong C-J. Association between renal function and cognitive performance in elderly community-dwelling men without dementia. J Am Geriatr Soc. 2010;58(10):2046-8.
42. Yao H, Miwa Y, Takashima Y, Yahara K, Hashimoto M, Uchino A, et al. Chronic kidney disease and subclinical lacunar infarction are independently associated with frontal lobe dysfunction in community-dwelling elderly subjects: the Sefuri brain MRI study. Hypertens Res Clin Exp. 2011;34(9):1023-8.
43. Zammit AR, Katz MJ, Lai JY, Zimmerman ME, Bitzer M, Lipton RB. Association between renal function and cognitive ability domains in the Einstein aging study: a cross-sectional analysis. J Gerontol A Biol Sci Med Sci. 2015;70(6):764-70.
44. Kurella M, Luan J, Yaffe K, Chertow GM. Validation of the Kidney Disease Quality of Life (KDQOL) cognitive function subscale. Kidney Int. 2004;66(6):2361-7.
45. Afsar B, Elsurer R, Covic A, Johnson RJ, Kanbay M. Relationship between uric acid and subtle cognitive dysfunction in chronic kidney disease. Am J Nephrol. 2011;34(1):49-54.
46. Afsar B. Relationship between total testosterone, cognitive function, depressive behavior, and sleep quality in chronic kidney disease patients not on dialysis. Clinical & Experimental Nephrology. 2013;17(1):59-65.
47. Arismendi-Morillo G, Fernandez-Abreu M. Ultrastructural cutaneous microvascular pathology of young adults aged up to 50 years with chronic kidney disease and vascular cognitive impairment. Ultrastruct Pathol. 2010;34(4):214-8.
48. Balasubramaniam K, Mawer GE, Pohl JE, Simons PJ. Impairment of cognitive function associated with hydroxyamylobarbitone accumulation in patients with renal insufficiency. Br J Pharmacol. 1972;45(2):360-7.
49. Barzilay JI, Gao P, O'Donnell M, Mann JFE, Anderson C, Fagard R, et al. Albuminuria and decline in cognitive function: The ONTARGET/TRANSCEND studies. Arch Int Med. 2011;171(2):142-150.
50. Buchman AS, Tanne D, Boyle PA, Shah RC, Leurgans SE, Bennett DA. Kidney function is associated with the rate of cognitive decline in the elderly. Neurology. 2009;73(12):920-7.
51. Corrao S, Argano C, Nobili A, Marucci M, Djade CD, Tettamanti M, et al. Brain and kidney, victims of atrial microembolism in elderly hospitalized patients? data from the REPOSI study. Eur J Intern Med. 2015;26(4):243-9.
52. Egbi OG, Ogunrin O, Oviasu E. Prevalence and determinants of cognitive impairment in patients with chronic kidney disease: a cross-sectional study in Benin City, Nigeria. Ann Afr Med. 2015;14(2):75-81.
53. Elias MF, Elias PK, Seliger SL, Narsipur SS, Dore GA, Robbins MA. Chronic kidney disease, creatinine and cognitive functioning. Nephrol Dial Transplant. 2009;24(8):2446-52.
54. Giallauria F, Fattirolli F, Tramarin R, Ambrosetti M, Griffo R, Riccio C, et al. Clinical characteristics and course of patients with diabetes entering cardiac rehabilitation. Diabetes Res Clin Pract. 2015;107(2):267-72.
55. Ginn HE, Teschan PE, Walker PJ, Bourne JR, Fristoe M, Ward FW, et al. Neurotoxicity in uremia. Kidney Int. 1975;7(3) S357-60.

56. Jassal SV, Roscoe J, LeBlanc D, Devins GM, Rourke S. Differential impairment of psychomotor efficiency and processing speed in patients with chronic kidney disease. Int Urol Nephrol. 2008;40(3):849-54.
57. Kang EW, Abdel-Kader K, Yabes J, Glover K, Unruh M. Association of sleep-disordered breathing with cognitive dysfunction in CKD stages 4-5. Am J Kidney Dis. 2012;60(6):949-58.
58. Kurella M, Chertow GM, Luan J, Yaffe K. Cognitive impairment in chronic kidney disease. J Am Geriatr Soc. 2004;52(11):1863-9.
59. Kurella Tamura M, Wadley V, Yaffe K, McClure LA, Howard G, Go R, et al. Kidney function and cognitive impairment in US adults: the Reasons for Geographic and Racial Differences in Stroke (REGARDS) Study. Am J Kidney Dis. 2008;52(2):227-34.
60. Kurella Tamura M, Muntner P, Wadley V, Cushman M, Zakai NA, Bradbury BD, et al. Albuminuria, kidney function, and the incidence of cognitive impairment among adults in the United States. Am J Kidney Dis. 2011;58(5):756-63.
61. Kurella Tamura M, Xie D, Yaffe K, Cohen DL, Teal V, Kasner SE, et al. Vascular risk factors and cognitive impairment in chronic kidney disease: the Chronic Renal Insufficiency Cohort (CRIC) study. Clin J Am Soc Nephrol. 2011;6(2):248-56.
62. Marchesi GF, Scarpino O, Fua P, Di Bella P, Salvolini U, Menichelli F, et al. Modificazioni neuropsichiche nella insufficienza renale cronica [Neuropsychic changes in chronic renal failure]. Riv Neurobiol. 1978;24(1-2):37-42.
63. Morosanu AI, Alexa ID, Badescu M, Ilie AC. Corelația între deficitul cognitive şi factorii de risc cardiovascular la pacientul vârstnic dializat vs. nedializat [Correlation between cognitive impairment and cardiovascular risk factors in dialysis vs. non-dialysis elderly patients]. Rev Med Chir Soc Med Nat Iasi. 2011;115(4):1057-1061.
64. Nulsen RS, Yaqoob MM, Mahon A, Stoby-Fields M, Kelly M, Varagunam M. Prevalence of cognitive impairment in patients attending pre-dialysis clinic. J Ren Care. 2008;34(3):121-6.
65. Post JB, Jegede AB, Morin K, Spungen AM, Langhoff E, Sano M. Cognitive profile of chronic kidney disease and hemodialysis patients without dementia. Nephron. 2010;116(3):c247-55.
66. Rabinowitz S, van der Spuy HIJ. Psychological adaptations to a renal dialysis and transplant program: a longitudinal and cross-section investigation. Int J Rehab Res. 1980;3(1):73-75.
67. Romijn MDM, van Marum RJ, Emmelot-Vonk MH, Verhaar HJJ, Koek HL. Mild chronic kidney disease is associated with cognitive function in patients presenting at a memory clinic. Int J Geriatr Psychiatry. 2015;30(7):758-65.
68. Sajjad I, Grodstein F, Kang JH, Curhan GC, Lin J. Kidney dysfunction and cognitive decline in women. Clin J Am Soc Nephrol. 2012;7(3):437-43.
69. Srivanitchapoom P, Senanarong V. Estimated creatinine clearance and cognitive impairment in Thai older adults: a pilot study from the dementia and disability project in Thailand. J Med Assoc Thai. 2013;96(2):S47-53.
70. Takuma T, Sanaka T, Sugino N. Intellectual impairment in chronic renal failure. Adv Exp Med Biol. 1987;223:131-4.
71. Terawaki H, Sato T, Miura N, Saito K, Mifune N, Shoji W, et al. [Assessment of competence of predialysis chronic kidney disease stage 5 patients to make treatment decisions: preliminary report]. Nippon Jinzo Gakkai Shi. 2008;50(7):915-26.
72. Teschan PE, Ginn HE, Bourne JR, Ward JW, Hamel B, Nunnally JC, et al. Quantitative indices of clinical uremia. Kidney Int. 1979;15(6):676-97.
73. Tryc AB, Alwan G, Bokemeyer M, Goldbecker A, Hcker H, Haubitz M, et al. Cerebral metabolic alterations and cognitive dysfunction in chronic kidney disease. Nephrol Dial Transplant. 2011;26(8):2635-41.
74. Williams UE, Owolabi MO, Ogunniyi A, Ezunu EO. Prevalence and pattern of neurocognitive impairment in Nigerians with stages 3 to 5 chronic kidney disease. ISRN Neurol. 2013;2013:374890.
75. Yamamoto Y, Ohara T, Nagakane Y, et al. Chronic kidney disease, 24-h blood pressure and small vessel diseases are independently associated

with cognitive impairment in lacunar infarct patients. Hypertens Res Clin Exp. 2011;34(12):1276-82.
