# Supplementary material for: Cognition in chronic kidney disease: a systematic review and meta-analysis
Source: BMC Med. 2016 Dec 14;14:206. doi: 10.1186/s12916-016-0745-9 (PMC5155375; doi:10.1186/s12916-016-0745-9)

Title: Cognition in chronic kidney disease: a systematic review and meta-analysis

Authors: I Berger, S Wu, P Masson, PJ Kelly, FA Duthie, W Whiteley, D Parker, D Gillespie, AC Webster

Additional File 3. Funnel Plots

Figure 1. eGFR 60 mL/min/1.73m^2 Orientation & Attention

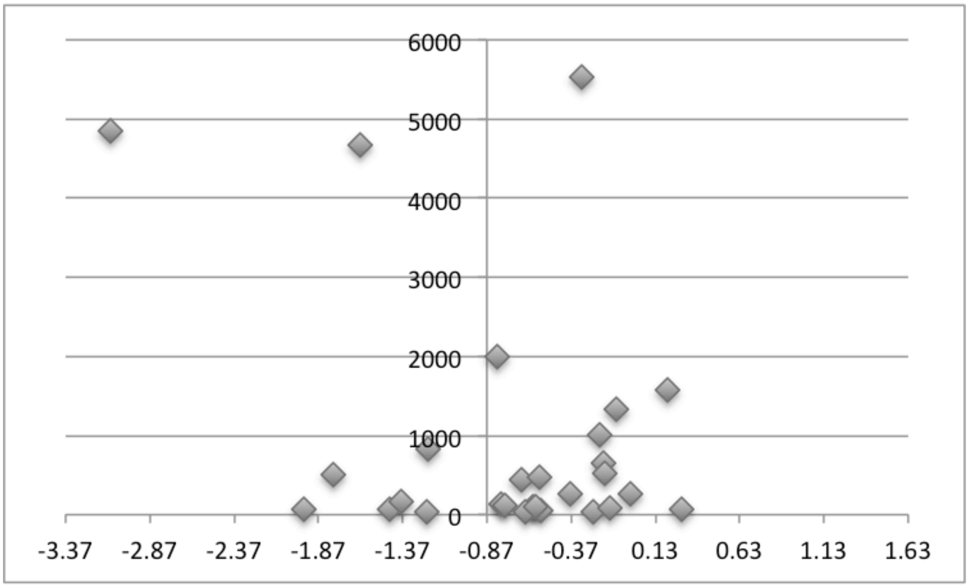

Figure 2. eGFR 60 mL/min/1.73m^2 Memory

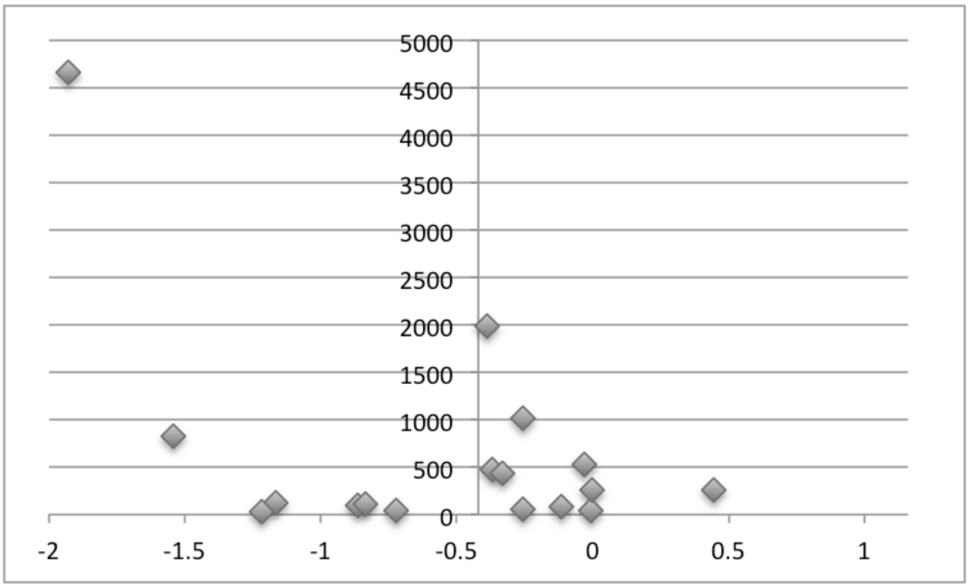

Title: Cognition in chronic kidney disease: a systematic review and meta-analysis

Authors: I Berger, S Wu, P Masson, PJ Kelly, FA Duthie, W Whiteley, D Parker, D Gillespie, AC Webster

Figure 3. eGFR 60 mL/min/1.73m^2 Executive Functions

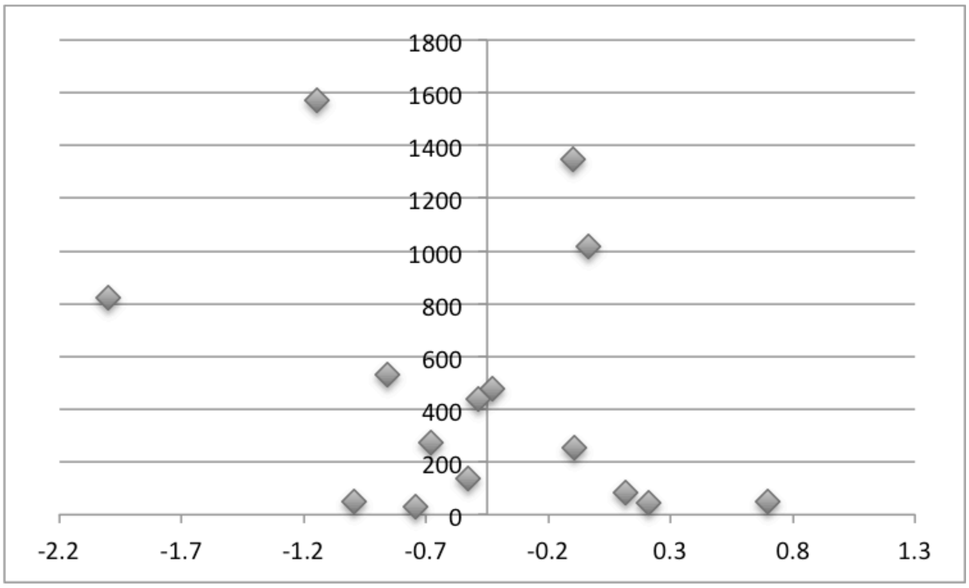

Figure 4. eGFR 60 mL/min/1.73m^2 Global Cognition

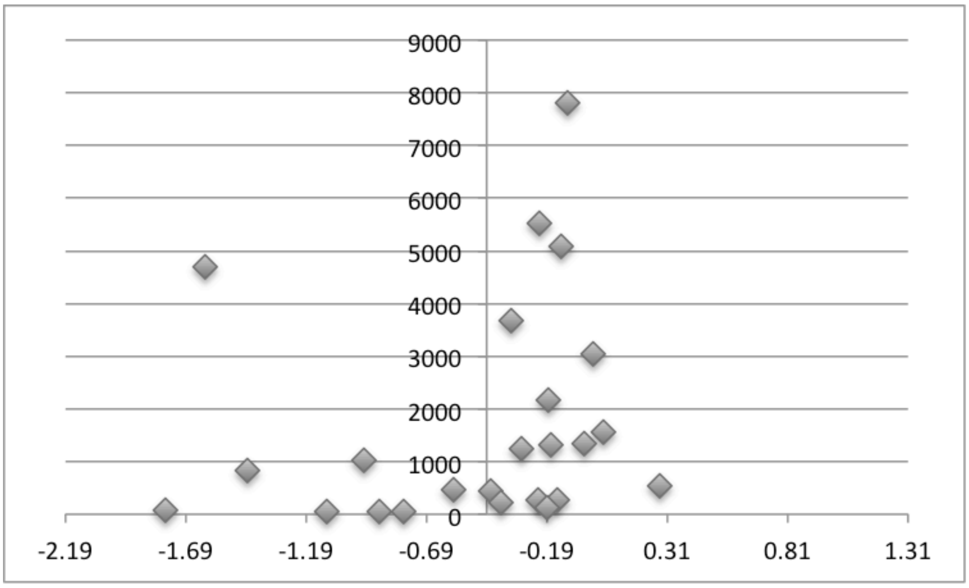

Title: Cognition in chronic kidney disease: a systematic review and meta-analysis

Authors: I Berger, S Wu, P Masson, PJ Kelly, FA Duthie, W Whiteley, D Parker, D Gillespie, AC Webster

Figure 5. eGFR 45 mL/min/1.73m^2 Global Cognition

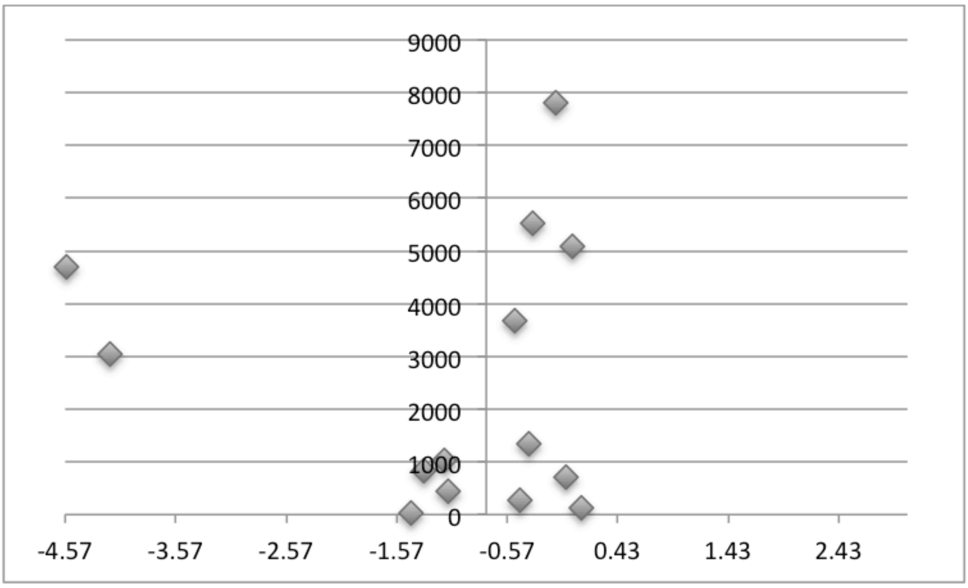

Figure 6. eGFR 30 mL/min/1.73m^2 Global Cognition

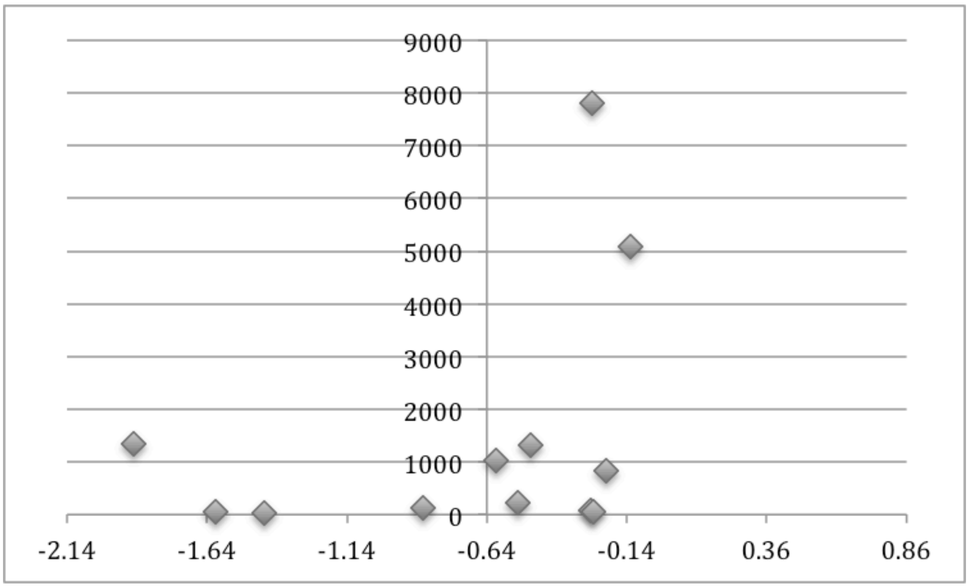

Supplement: Additional file 3: — Funnel plots. (PDF 259 kb) [file 12916_2016_745_MOESM3_ESM.pdf]
